# Supplementary material for: Current-driven dynamics and inhibition of the skyrmion Hall effect of ferrimagnetic skyrmions in GdFeCo films
Source: Nat Commun. 2018 Mar 6;9:959. doi: 10.1038/s41467-018-03378-7 (PMC5840382; doi:10.1038/s41467-018-03378-7)
Supplement: Supplementary file 1 — Supplementary Information [file 41467_2018_3378_MOESM1_ESM.pdf]

Current-driven dynamics and inhibition of the skyrmion Hall effect of ferrimagnetic skyrmions in GdFeCo films

Woo et al.

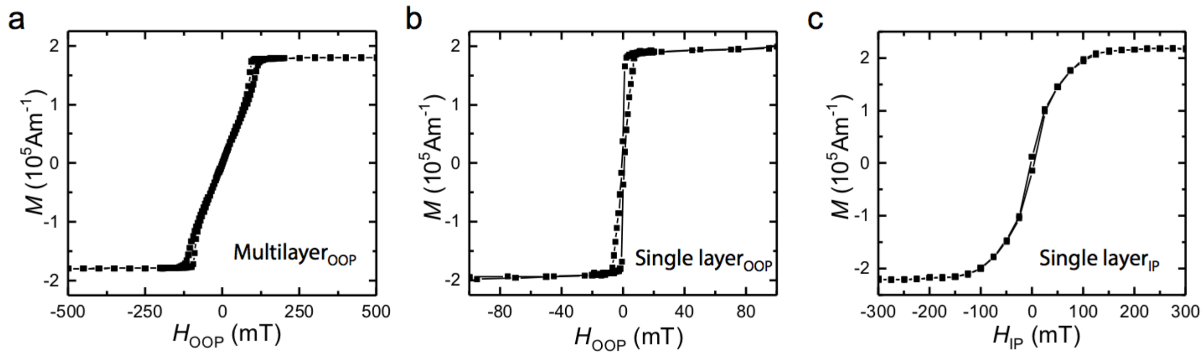

Supplementary Figure 1. Hysteresis loops of our Pt/GdFeCo/MgO films. (a) Easy-axis out-of-plane hysteresis loops for a companion multilayer film grown on SiO_x/Si wafer. **(b)** Out-of-plane and **(c)** in-plane hysteresis loop for the unit structure, single layer Pt/GdFeCo/MgO film grown on Si wafer. The saturation magnetic moment is acquired to be $M_S = 2\times10^5$ A m^{-1}.

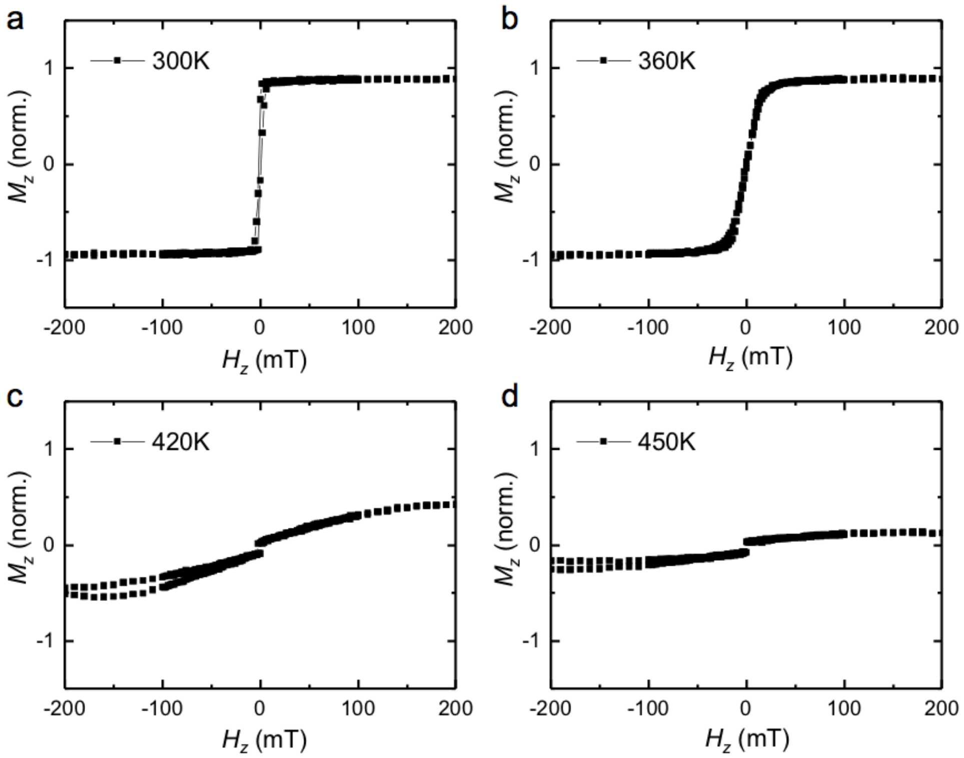

Supplementary Figure 2. Easy axis hysteresis loops as a function of temperature. Out-of-plane hysteresis loops for a Pt/CoFeB/MgO unit layer grown on SiO_x/Si wafer measured at **(a)** T = 300 K, **(b)** T = 360 K, **(c)** T = 420 K, **(d)** T = 450 K, respectively. The measurement was conducted using vibrating sample magnetometry (VSM) with the accessible temperature range of 100 ~ 450 K.

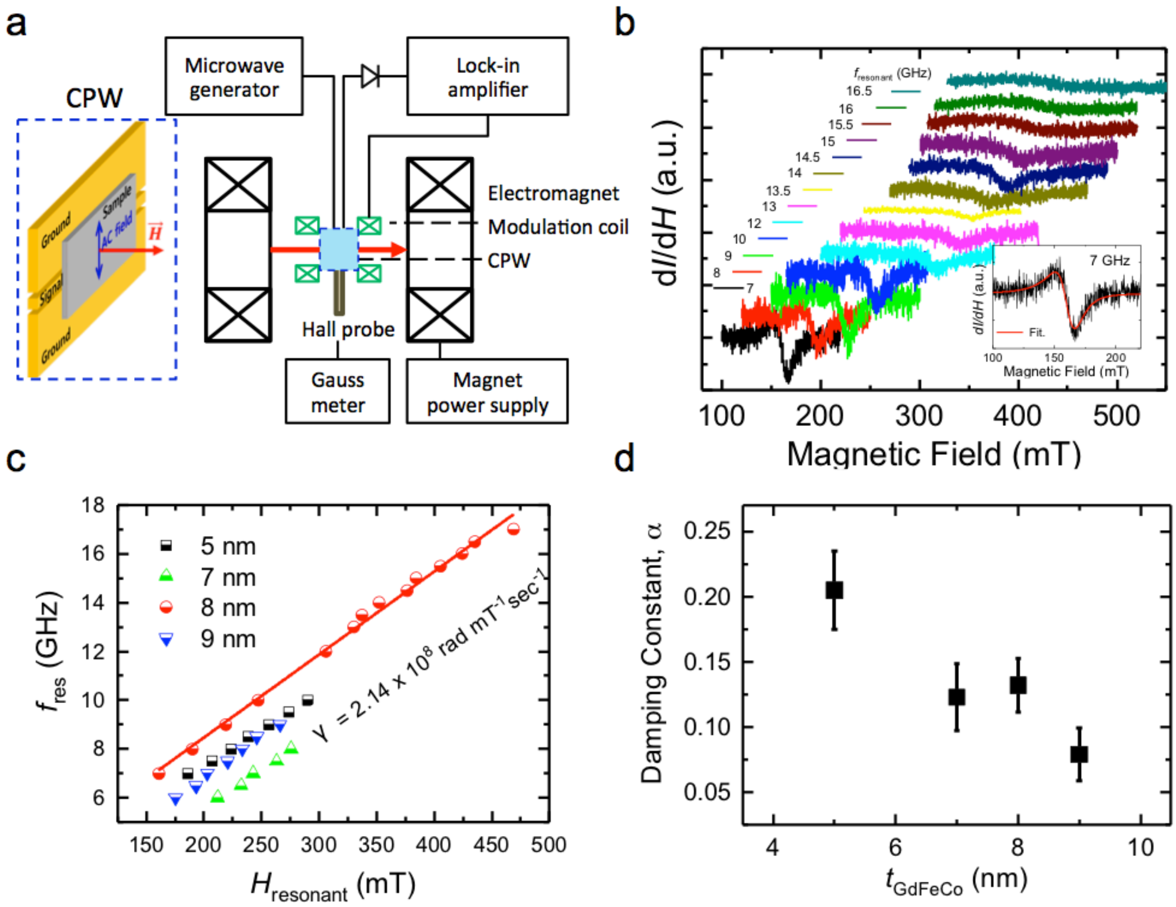

Supplementary Figure 3. Ferromagnetic resonance (FMR) for the measurement of the effective damping constant. (a) Configuration of experimental set-up for FMR measurement. Note that the magnetic field was applied along the out-of-plane direction during the measurements. **(b)** FMR absorption spectra at frequencies between 7 and 16.5 GHz in a sample of t_{GdFeCo} = 8 nm. **(c)** The external magnetic field-dependent resonant frequency (f_{res}) variation as a function of GdFeCo thickness in Pt(3 nm)/GdFeCo(t_{GdFeCo} nm)/MgO(1 nm) unit structure. Fitting to Eq. (1) shown in Supplementary Note 3 yields the gyromagnetic ratio (γ) for each structure, and the gyromagnetic ratio for all structures turns out to be similar, ~2.14×10^{8} rad $\mathrm{mT^{-1} s^{-1}}$. **(d)** Calculated effective damping constant as a function of GdFeCo thickness. By fitting the experimental data of each t_{GdFeCo} to Eq. (2), we obtain the effective damping constant of α = 0.205 ± 0.035 for t_{GdFeCo} = 5 nm. Error bars denote the standard deviation of multiple measurements.

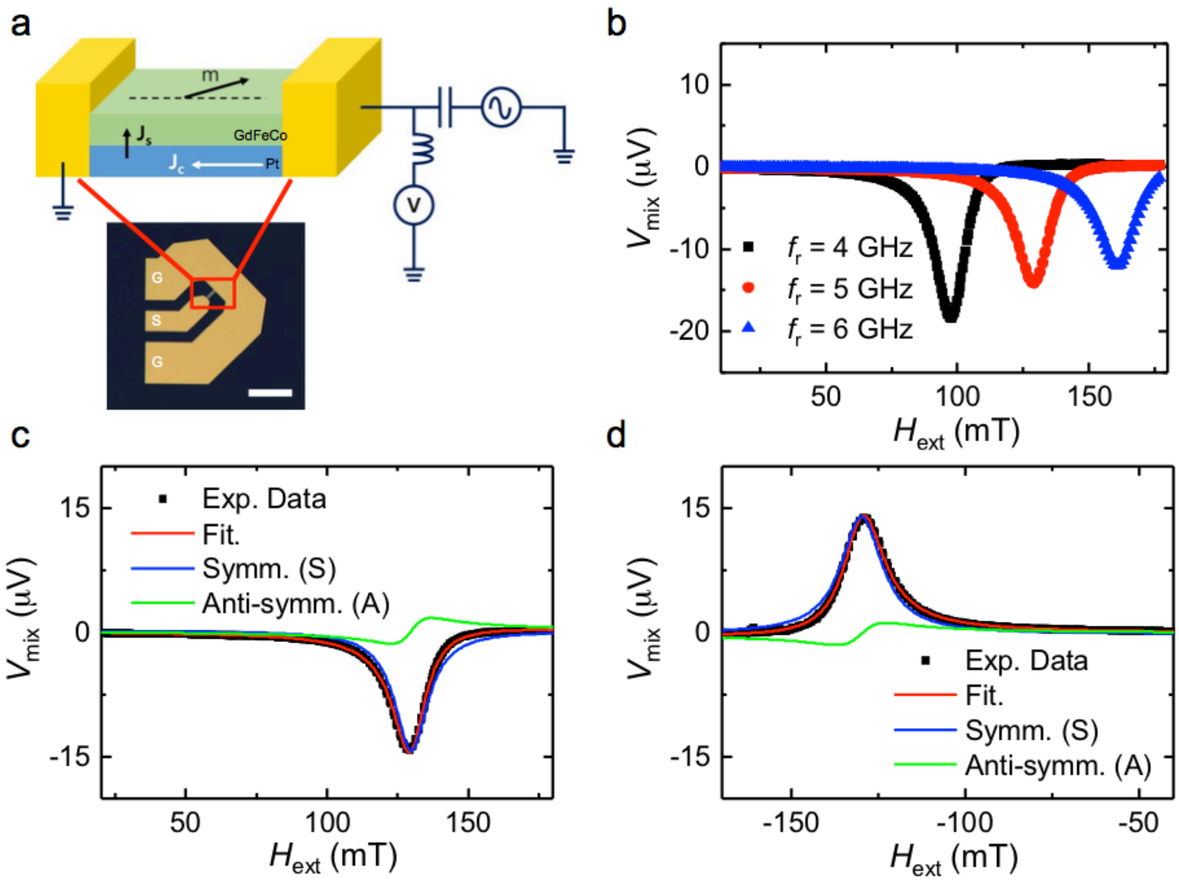

Supplementary Figure 4. Spin-torque ferromagnetic resonance (ST-FMR) for the measurement of the effective spin Hall angle. (a) Schematic description of a device and the measurement configuration. In-plane charge current and resulting out-of-plane spin current via spin Hall effect are denoted as j_c and j_s, respectively. The optical microscope image of the actual sample and electrical contacts are also shown. Scale bar, 100 μm. **(b)** ST-FMR signals for various resonant frequencies, f_r = 4-6 GHz. Note that resonant field shifts to a larger value at higher f_r, reflecting the nature of ST-FMR measurement. Experimental ST-FMR signals (dot) at **(c)** positive and **(d)** negative external fields, and the fitting of the symmetric Lorentzian and asymmetric Lorentzian functions to the data using the Eq. (3) and Eq. (4).

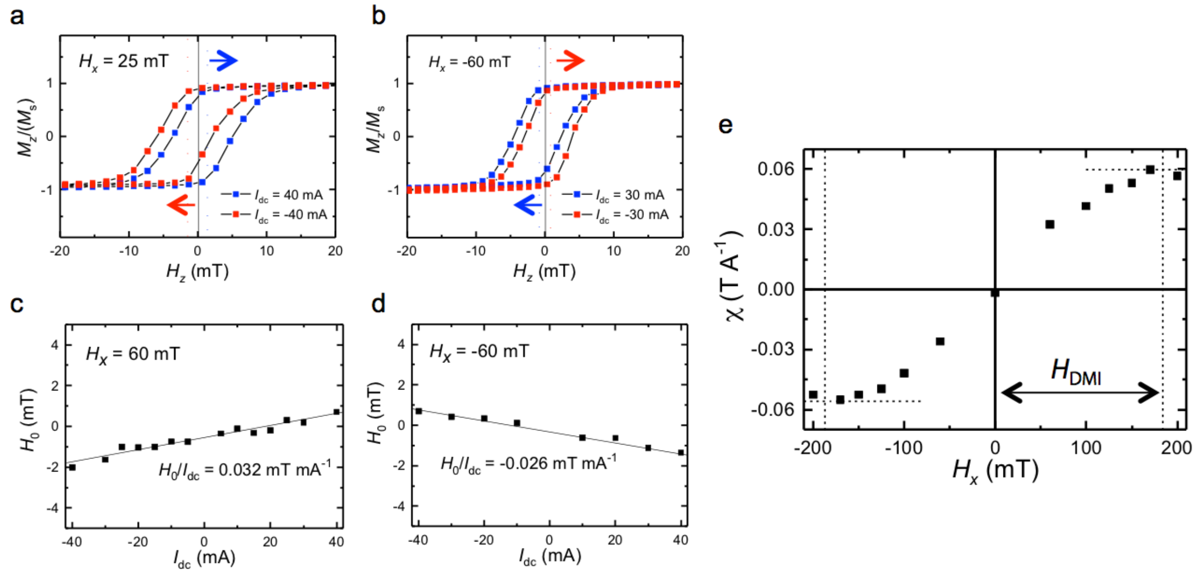

Supplementary Figure 5. Electrical measurement of Dzyaloshinskii-Moriya interaction (DMI) magnitude. (a), **(b)** Anomalous Hall loops under dc-currents I_{dc} = ±40 mA (I_{dc} = ±30 mA) and an in-plane field H_x = 25 mT (H_x = -60 mT). **(c), (d)** Variation of the center of hysteresis loop, H_o, as a function of the amount of in-plane current at H_x = 60 mT and H_x = -60 mT, respectively. For each figure, the spin Hall effective field per unit current in mA is calculated and shown. **(e)** The measured effective χ, the amount of hysteresis loop shift per unit current in T/A, as a function of H_x. The estimate of DMI effective field, H_{DMI}, is indicated.

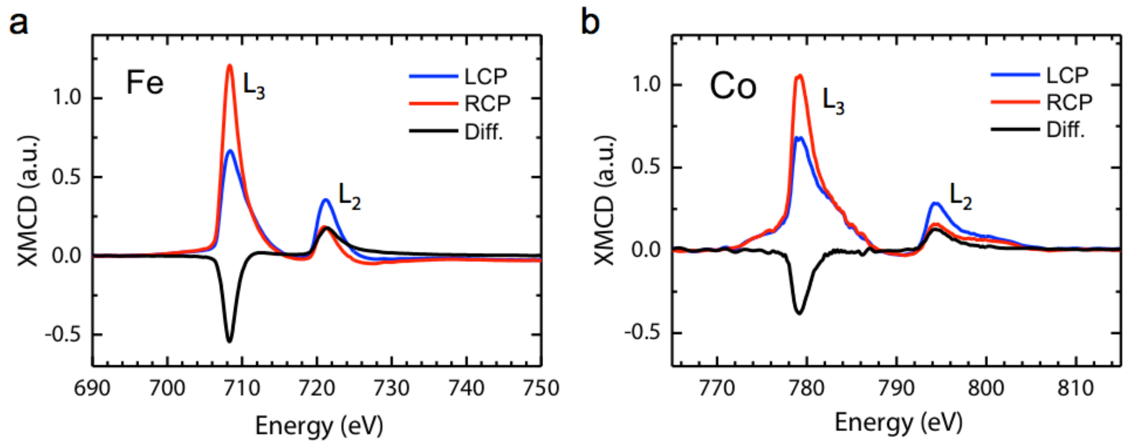

Supplementary Figure 6. X-ray magnetic circular dichroism spectroscopy (XMCD) measurement. The XMCD spectra at the L_3 and L_2 edges of **(a)** Fe and **(b)** Co, respectively. Note that the XMCD spectra from right-circular-polarized (RCP) left-circular-polarized (LCP) X-rays are shown.

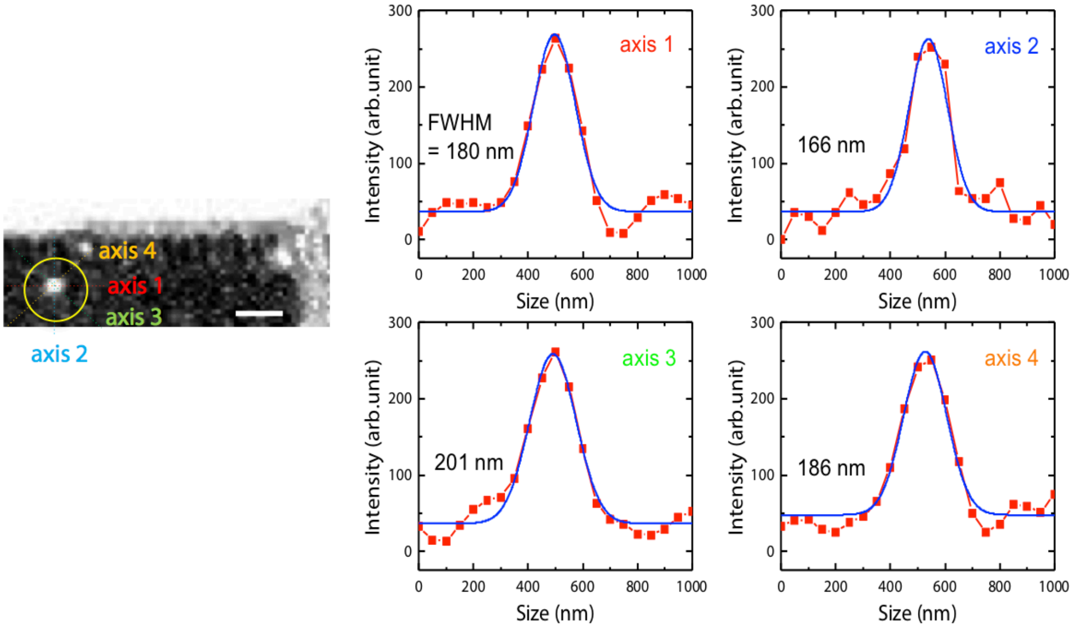

Supplementary Figure 7. Skyrmion diameter measurement. A representative enlarged scanning transmission X-ray microscopy (STXM) image showing a skyrmion shown in Fig. 2 in the main text. A single skyrmion with four lines crossing the center of the skyrmion. Lines are drawn along the direction $y = 0$ (axis 1), $x = 0$ (axis 2), $y = -x$ (axis 3) and $y = x$ (axis 4) and used for contrast line-scans. The contrast line scans with its Gaussian fitting and FWHM, which are used to determine the diameter of skyrmions, are shown on the right side of the STXM image. Scale bar, 500 nm.

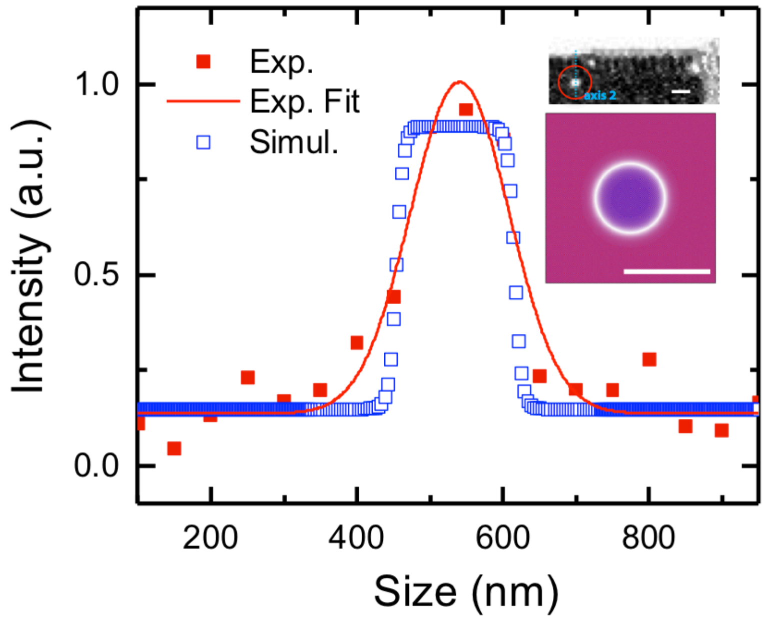

Supplementary Figure 8. Experimental and simulated profiles of the ferrimagnetic skyrmion. The experimental data are adopted from Supplementary Fig. 7 (axis 2), where the external magnetic field of H_z = -145 mT is applied. The simulation is performed in a 4000 Å × 4000 Å square film with the cell size of 20 Å. The simulation parameters are: $A_{\text{Gd-Fe}}$ = -10 pJ m^{-1}, D = -0.96 mJ m^{-2}, K_u = 4.01×10^4 J m^{-3}, M_S = 2×10^5 A m^{-1}, and n = 0.78. Note that, for the purpose of correctly comparing the skyrmion diameter and profile, the simulated skyrmion profile is not rescaled. An external out-of-plane magnetic field (H_z) of -140 mT is applied in the simulation. Insets show the experimental skyrmion image, taken at H_z = -145 mT, used for the comparison and simulated ground-state skyrmion snapshot at H_z = -140 mT. Scale bars, 200 nm. It is worth mentioning that our skyrmion has an ~ 180 nm diameter while the domain wall width is typically about ~10 nm ($\sqrt{A/K_{\text{u,eff}}}$). Therefore, the simulation profile looks like a so-called intermediate-size bubble skyrmion with a large skyrmion core and sharp domain wall width, while the sharp domain walls (the sharp transition from background to skyrmion core) is not shown in experiments, due to the spatial resolution of X-ray measurement is limited to ~25 nm.

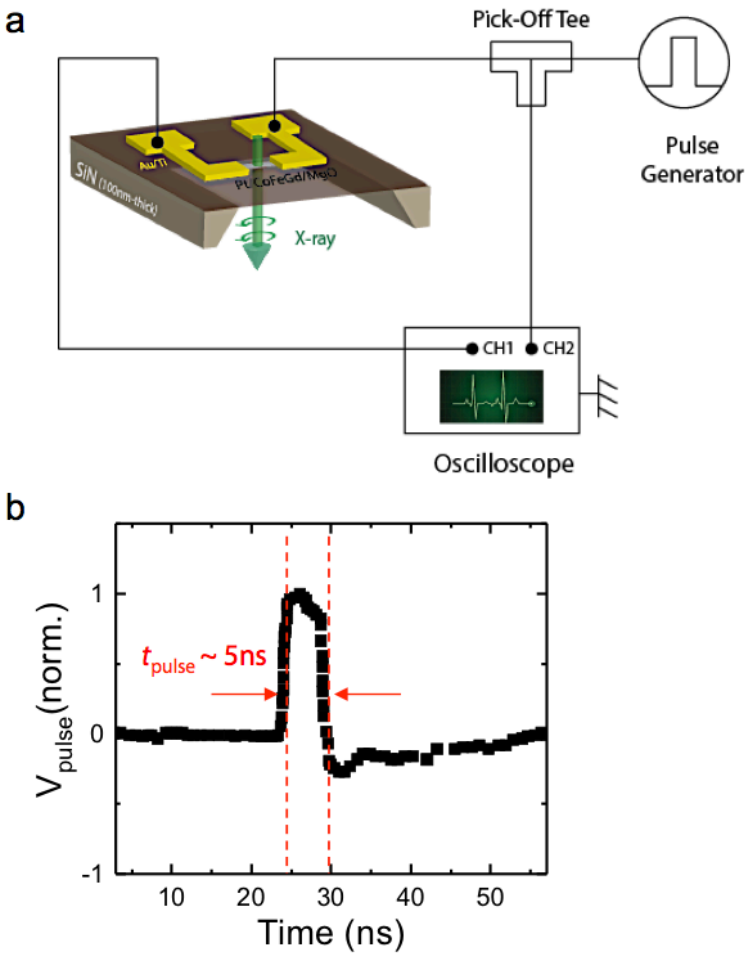

Supplementary Figure 9. Circuit diagram and an example of electrical pulse used for skyrmion excitation measured by oscilloscope. (a) Schematic of the electric connection for current pulse-injection experiments at scanning transmission X-ray microscopy (STXM) beamline. The X-ray beam is injected at the frequency of 500 MHz (bunch spacing = 2 ns). A -20 dB pickoff T is connected to simultaneously observe transmitted and reflected pulses. **(b)** An exemplary 5-ns-long pulse used for excitations shown in the main text Fig. 2.

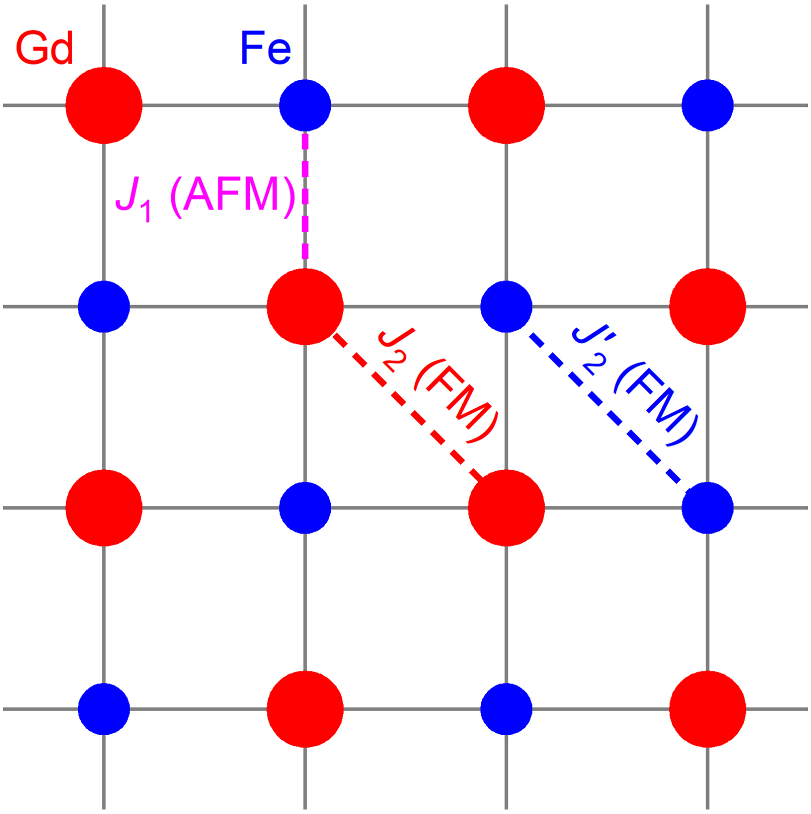

Supplementary Figure 10. The checkerboard-like two-sublattice spin system based on a square lattice including both antiferromagnetic (AFM) inter-sublattice (J_1) and ferromagnetic (FM) intra-sublattice (J_2, J_2') Heisenberg exchange interactions. The AFM J_1, FM J_2, and FM J_2' exchange interactions are indicated by dashed lines. Note that $J_1 = aA_1$, $J_2 = \sqrt{2}aA_2$, $J_2' = \sqrt{2}aA_2'$, where A_i denotes the exchange coefficient used in the simulation and a stand for the lattice constant.

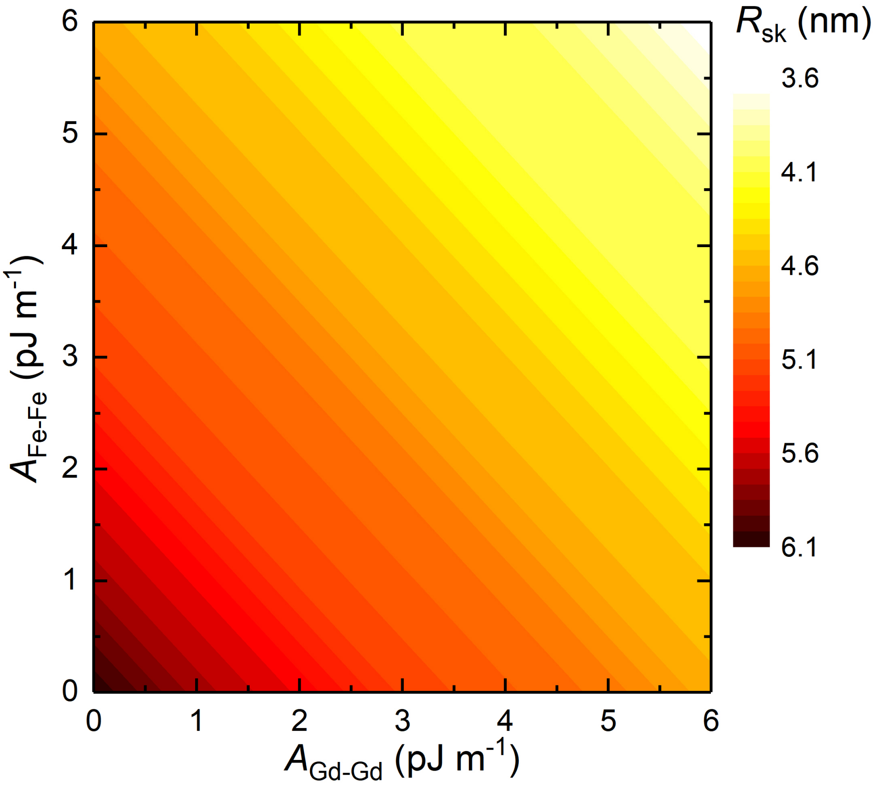

Supplementary Figure 11. Static property of a ferrimagnetic skyrmion in the model with both antiferromagnetic inter-sublattice and ferromagnetic intra-sublattice exchange interactions. The color contour indicates the skyrmion radius as functions of the Gd-Gd and Fe-Fe intra-sublattice exchange interactions. The simulation is performed in a 750 Å × 750 Å film with periodic boundary conditions (PBCs) in both x and y directions and the lattice constant of 5 Å. The simulation parameters are: $A_{\mathrm{Gd\text{-}Fe}}$ = -10 pJ m^{-1}, $A_{\mathrm{Gd\text{-}Gd}}$ = 0~6 pJ m^{-1}, $A_{\mathrm{Fe\text{-}Fe}}$ = 0~6 pJ m^{-1}, D = -0.96 mJ m^{-2}, K_u = 4.01×10^4 J m^{-3}, M_S = 2×10^5 A m^{-1}, and n = 0.78.

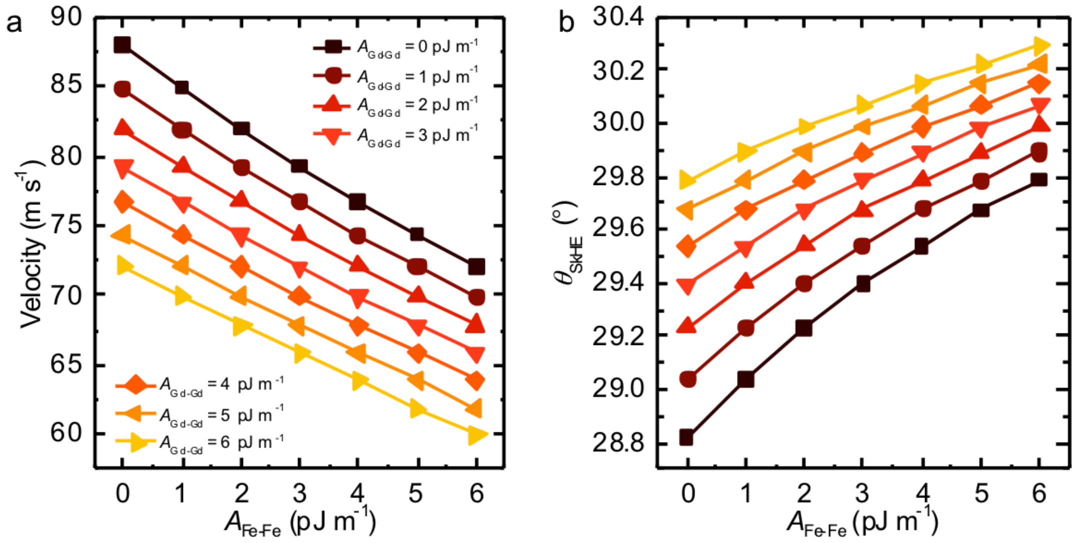

Supplementary Figure 12. Dynamic property of a ferrimagnetic skyrmion in the model with both antiferromagnetic inter-sublattice and ferromagnetic intra-sublattice exchange interactions. (a) The skyrmion velocity as functions of the Gd-Gd and Fe-Fe intra-sublattice exchange interactions. **(b)** The skyrmion Hall angle as functions of the Gd-Gd and Fe-Fe intra-sublattice exchange interactions. The simulation is performed in a 750 Å × 750 Å film with periodic boundary conditions (PBCs) in both *x* and *y* directions and the lattice constant of 5 Å. The simulation parameters are: $A_{\text{Gd-Fe}}$ = -10 pJ m^{-1}, $A_{\text{Gd-Gd}}$ = 0~6 pJ m^{-1}, $A_{\text{Fe-Fe}}$ = 0~6 pJ m^{-1}, D = -0.96 mJ m^{-2}, K_u = 4.01×10^4 J m^{-3}, M_S = 2×10^5 A m^{-1}, j_a = 5×10^{11} A m^{-2}, α = 0.205, θ_{SH} = 0.055, and n = 0.78.

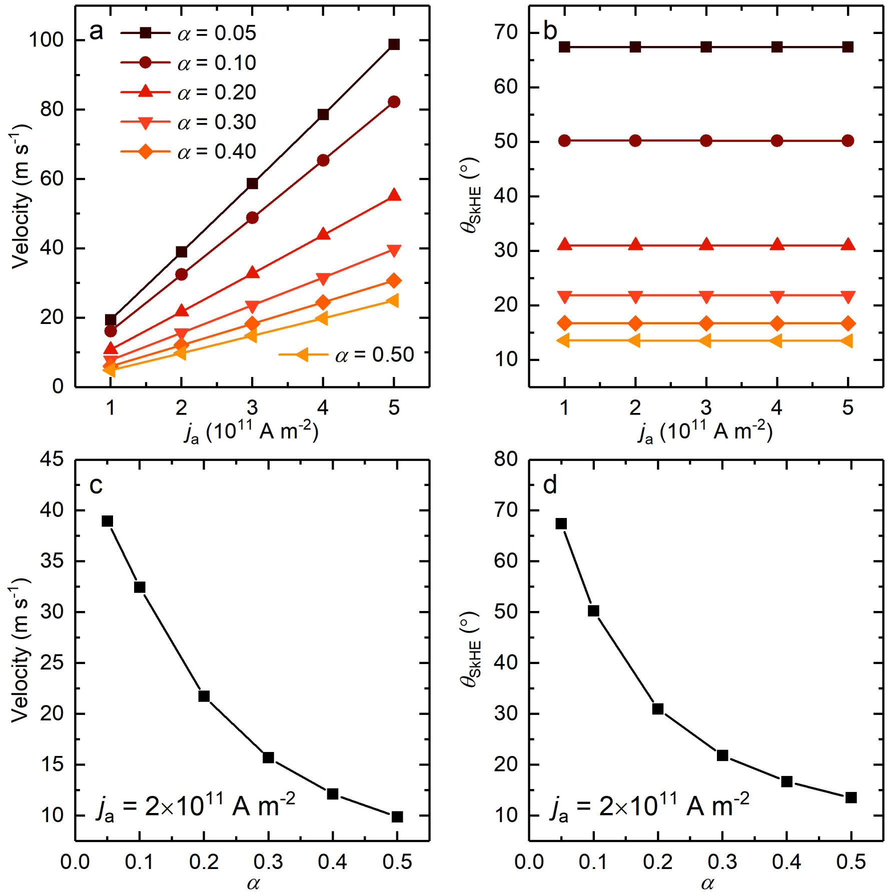

Supplementary Figure 13. Current-driven motion of a ferrimagnetic skyrmion in models with different damping coefficients. (a) The skyrmion velocity as a function of the driving current density for different values of the damping coefficient. **(b)** The skyrmion Hall angle as a function of the driving current density for different values of the damping coefficient. **(c)** The skyrmion velocity as a function of the damping coefficient. **(d)** The skyrmion Hall angle as a function of the damping coefficient. The simulations are performed in a 750 Å × 750 Å film with periodic boundary conditions (PBCs) in both x and y directions and the lattice constant of 5 Å. The simulation parameters are: $A_{\text{Gd-Fe}}$ = -10 pJ m^{-1}, D = -0.96 mJ m^{-2}, $K_u = 4.01\times10^4$ J m^{-3}, $M_S = 2\times10^5$ A m^{-1}, B_z = 130 mT, θ_{SH} = 0.055, and n = 0.78.

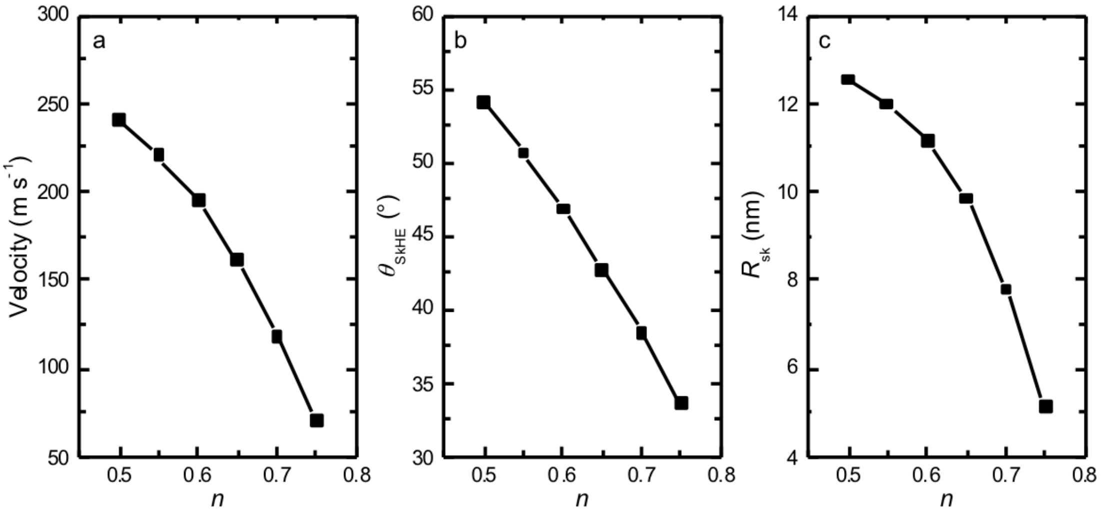

Supplementary Figure 14. Current-driven motion of a ferrimagnetic skyrmion in models with different compensation ratio, *n*. (a) The skyrmion velocity as a function of *n*. **(b)** The skyrmion Hall angle as a function of *n*. **(c)** The skyrmion radius as a function of *n*. The simulations are performed in a 750 Å × 750 Å film with periodic boundary conditions (PBCs) in both *x* and *y* directions and the lattice constant of 5 Å. The simulation parameters are: $A_{Gd\text{-}Fe}$ = -10 pJ m^{-1}, D = -0.96 mJ m^{-2}, K_u = 4.01×10^4 J m^{-3}, M_S = 2×10^5 A m^{-1}, B_z = 130 mT, α = 0.205, θ_{SH} = 0.055, and j_a = 4×10^{11} A m^{-2}.

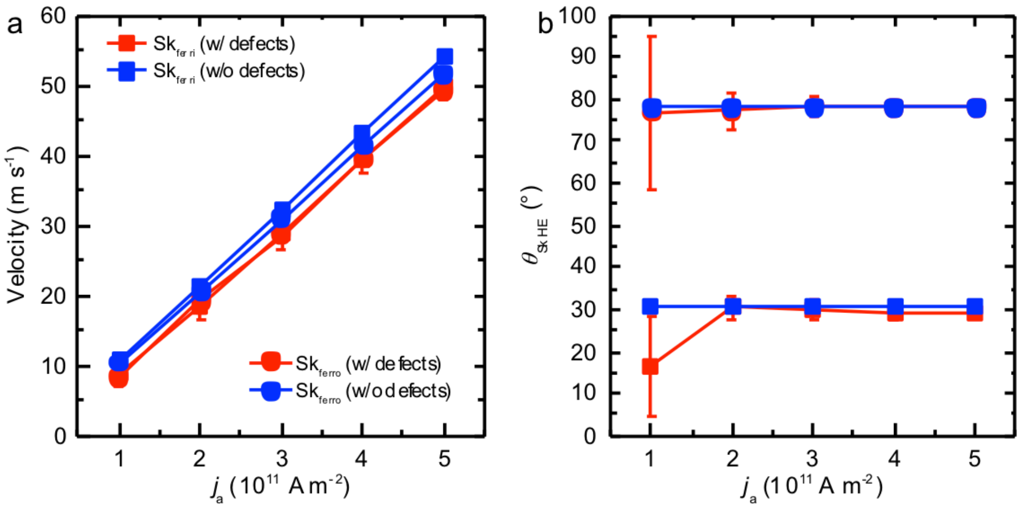

Supplementary Figure 15. Current-driven motion of ferrimagnetic and ferromagnetic skyrmions in the same clean and disorder models. (a) The skyrmion velocity as a function of the driving current density. **(b)** The skyrmion Hall angle as a function of the driving current density. The simulations are performed in a 750 Å × 750 Å film with periodic boundary conditions (PBCs) in both x and y directions and the lattice constant of 5 Å. The simulation parameters for the ferrimagnetic skyrmion are: $A_{\text{Gd-Fe}}$ = -10 pJ m^{-1}, D = -0.96 mJ m^{-2}, $K_u = 4.01\times10^4$ J m^{-3}, $M_S = 2\times10^5$ A m^{-1}, B_z = 130 mT, α = 0.205, θ_{SH} = 0.055, and n = 0.78. The simulation parameters for the ferromagnetic skyrmion are: A = 10 pJ m^{-1}, D = 0.96 mJ m^{-2}, $K_u = 4.01\times10^4$ J m^{-3}, $M_S = 2\times10^5$ A m^{-1}, B_z = 900 mT, α = 0.205, and θ_{SH} = 0.055. The sizes of the ferrimagnetic and ferromagnetic skyrmions are basically identical. In both the ferrimagnetic and ferromagnetic disorder models, the parameters for the randomly distributed pinning sites are the same: pinning site size is set as 2 × 2 spins, pinning site density is equal to 20 %, $K_{\text{pinning}} = 5K_u$.

Supplementary Note 1. Hysteresis loops of Pt/GdFeCo/MgO single and multilayer films.

Supplementary Fig. 1a presents easy-axis hysteresis behaviour of our Pt/GdFeCo/MgO multilayer, measured by vibrating sample magnetometry (VSM), used in the main text. It should be noted that the vanishing remnant magnetization in the multilayer structure is due to the formation of multi-domain states in the absence of magnetic field, which results from the strong de-magnetizing field. This is confirmed by the scanning transmission X-ray microscopy (STXM) magnetic domain images shown in the main text Fig. 1a, where dark and bright contrasts correspond to down and up domains, respectively. Supplementary Fig. 1b and 1c show the out-of-plane and in-plane hysteresis loops of unit structure, single-layered Pt/GdFeCo/MgO, respectively. From these hysteresis loops of unit layer, we confirm the material parameters: saturation magnetization being M_S = 2×10^5 A m^{-1} and uniaxial anisotropy constant being $K_u = 4.01\times10^4$ J m^{-3}.

Supplementary Note 2. Magnetization compensation temperature measurement.

In ferrimagnetic materials such as GdFeCo, there exists a magnetization compensation temperature, T_M, where the magnetic moments of Gd and FeCo are cancelled, M_{S_FeCo} = M_{S_Gd}, resulting in zero net magnetization. Because the skyrmion Hall effect is linearly proportional to the topological charge as well as net magnetization, skyrmions behave like antiferromagnetic skyrmions only near the compensation temperature by completely suppressing the skyrmion Hall effect.[1–3]

Supplementary Fig. 2 presents the temperature-dependent out-of-plane magnetization hysteresis loops of our Pt/GdFeCo/MgO unit structure grown on SiO_x/Si substrate, measured by VSM. The magnetic moment approaches to zero at ~450 K. We were not able to measure the hysteresis loops for temperatures higher than 450 K due to the temperature limitation of our VSM machine. Therefore, we can only note that the compensation temperature of our GdFeCo film is slightly above 450 K, which also agrees well with earlier report Ref. [4]. It should also be noted that the GdFeCo structure remains at uncompensated ferrimagnetic state at room temperature, where the current-driven skyrmion dynamics were measured in the main text.

Supplementary Note 3. Measurement of material parameters

Using the single-layer companion Pt/GdFeCo/MgO film grown on SiO_x/Si wafer, which were shown in Supplementary Fig. 1 and Supplementary Note 1, we have performed several measurements to quantify key material parameters; magnetic damping, spin Hall angle, DMI magnitude and magnetization compensation ratio. Below, we provide measurement details and obtained parameters.

- **Magnetic damping constant**

Conventional ferromagnetic resonance (FMR)[5] measurement was performed at room temperature using a flip-chip method.[6] As will be discussed in Supplementary Note 7, the ferrimagnetic skyrmion dynamics is highly sensitive to small changes in damping constant. Therefore, we carefully performed damping constant measurements for slightly-different GdFeCo effective thickness samples, t_{GdFeCo} = 5~9 nm, and measured multiple samples for the same thickness to have large enough statistics for extracting accurate value. Supplementary Fig. 3a shows the schematic configuration of FMR spectrometer, in which *unpatterned* sample was loaded onto the coplanar waveguide (CPW) center and the magnetic field (H) was applied perpendicular to the plane. Field-swept FMR signal was obtained as a function of the microwave frequency. We employed field modulated lock-in technique using modulation coil to enhance the signal to noise ratio (SNR).

Supplementary Fig. 3b presents an exemplary series of field swept absorption spectra at frequencies between 7 and 16.5 GHz for Pt/GdFeCo/MgO film of t_{GdFeCo} = 8 nm. As the frequency (f) increases, the resonant peak position shifts to the higher magnetic field and the peak broadens. The resonance field ($H_{resonant}$) and the spectral linewidth were obtained by fitting the absorption data to the sum of symmetric and asymmetric derivative Lorentzian curve (Supplementary Fig. 3b, inset).[7] The frequency dependence of H_{res} is plotted in Supplementary Fig. 3c. By fitting the results at various GdFeCo thicknesses, the average gyromagnetic ratio (γ) can be determined from the resonance condition for perpendicular geometry (Kittel equation, see Eq. (1))[8]

$$f_{\perp} = \left(\frac{\gamma}{2\pi}\right)\left[H_{\mathrm{resonant}} - 4\pi M_s + \frac{2K_1}{M_s}\right] \quad (1)$$

where M_s is the saturation magnetization and K_1 is the effective uniaxial anisotropy. The calculated gyromagnetic ratios are very similar for all GdFeCo samples, as $\gamma \sim 2.14 \times 10^8$ rad $\mathrm{mT^{-1}s^{-1}}$. We also plotted the frequency-dependent full width at half maximum

(FWHM, ΔH) at each GdFeCo thickness, where ΔH values were calculated from the peak-peak line width (ΔH_{pp}) of the derivative curve using the relation of $\Delta H = \sqrt{3}\,\Delta H_{pp}$.[9] The ΔH is proportional to the frequency in the following equation, Eq. (2),[8]

$$\Delta H(f) = \Delta H_0 + \frac{4\pi\alpha}{\gamma} f \tag{2}$$

where the ΔH_0 is the inhomogeneous linewidth broadening, which can be related to the sample inhomogeneity. Finally, the magnetic damping parameter can be extracted from the slope of the each ΔH curve, and Supplementary Fig. 3d plots the calculated damping parameter for various GdFeCo thicknesses, t_{GdFeCo} = 5~9 nm. It is observed that the effective damping constant increases from α = 0.079 ± 0.02 for t_{GdFeCo} = 9 nm to α = 0.205 ± 0.035 for t_{GdFeCo} = 5 nm, and this trend is reasonable as the effective spin pumping into the Pt bottom layer increases as the thickness of magnetic layer becomes thinner.

Because the thickness of GdFeCo is 5 nm in the samples used for skyrmion experiments, we have used the damping coefficient of α = 0.205 ± 0.035 for micromagnetic simulations.

- **Spin Hall angle**

In order to evaluate the effective spin Hall angle, θ_{SH}, we performed spin-torque ferromagnetic resonance (ST-FMR) measurements. We fabricated devices by patterning Pt/GdFeCo/MgO thin-films into micrometer-sized rectangular structures, and subsequently depositing Ti(5)/Au(100) waveguides for electrical contacts. Supplementary Fig. 4a shows the measurement scheme, where the optical microscope image of a device is included. A microwave electrical current at fixed frequency (3.5 to 6.5 GHz) was applied to the devices while sweeping the magnetic field (H_{ext}), from 0 to 180 mT. The radio-frequency (RF) current in Pt generates spin-orbit torques and Oersted field torque to the adjacent ferrimagnetic layer, and when the frequency and the external field satisfy FMR condition, the resonant magnetization precession within GdFeCo layer can be excited. During the resonance, the precessional motion of the magnetization produces the oscillation of electrical resistance due to the oscillating anisotropic magnetoresistance. The mixed DC voltage generated across the device can be decoupled from mixed RF signal by using bias tee, and the voltage can then be fitted by the sum of symmetric Lorentzian and asymmetric Lorentzian functions,[10]

$$V_{mix}(H) = S\frac{\Delta H^2}{(H_{ext}-H_o)^2+(\Delta H)^2} + A\frac{(H_{ext}-H_o)\Delta H}{(H_{ext}-H_o)^2+\Delta H^2} \tag{3}$$

where S and A are the voltage amplitudes of symmetric and asymmetric Lorentzian function, respectively, H_o is the resonance field, and ΔH is the resonance linewidth. The value of S corresponds to the anti-damping torque while the one of A to the field-like and Oersted torques.[10] Supplementary Figs. 4b-d presents exemplary ST-FMR signals and S and A fittings. The spin Hall angle can be quantified from the ratio analysis, on the assumption that the field-like component is small, that is expressed as Eq. (4),[10]

$$\theta_{SH} = e\frac{J_s}{J_c} = \frac{S}{A}\frac{e\mu_o M_s t_{Pt} t_{GdFeCo}}{\hbar}\sqrt{1+\frac{M_{eff}}{H_o}} \tag{4}$$

where M_{eff} is the effective demagnetization field, t_{Pt} is the thickness of Pt and t_{GdFeCo} is the thickness of GdFeCo. The magnitude of M_{eff} was estimated by using Kittel's formula and the *M-H* hysteresis behavior of the film, where both estimations resulted in a consistent M_{eff} value, M_{eff}~7 mT. Finally, using Eq. (4) and measured material parameters & relevant constants, we have estimated the effective angle, θ_{SH} being 0.055 ± 0.007.

- **DMI magnitude**

In order to measure the magnitude of DMI, we utilized the electrical measurement technique developed by Pai et al., in Ref. [11]. We first patterned the GdFeCo film into 120 μm-wide Hall bar structure and measured the anomalous Hall (AH) voltages to evaluate its magnetization state.

In this technique, we measure the out-of-plane magnetic field (H_z)-driven hysteresis loop shifts under the simultaneous application of in-plane magnetic field (H_x) and in-plane dc-current I_{DC}. In case of homochiral Néel domain walls (DWs), which are expected in our GdFeCo multilayers, strong in-plane magnetic field can break the symmetry between *up-to-down* and *down-to-up* Néel DWs, thus, the in-plane dc-current, which injects pure vertical spin current via spin Hall effect (SHE), acts differently on two DWs. Note that that an average of spin moments in Néel DWs changes from <cosΦ = 0> (equal number of DWs antiparallel or parallel to H_x) to <cosΦ = 1> (all DWs parallel to H_x) when H_x approaches to H_{DMI},[12] where Φ being the angle between spin moment in DWs and *x*-axis (in-plane field direction). Therefore, the amount of hysteresis loop shift per unit current, labeled as the spin Hall effect efficiency χ_{SHE}, increases until the in-plane field reaches the effective DMI field ($H_{\mathrm{x}} = H_{\mathrm{DMI}}$), where there exists the largest asymmetry between *up-to-down* and *down-to-up* DWs, and then saturates. By quantifying the in-plane field where χ_{SHE} saturates, we can calculate the DMI effective field, H_{DMI}, which can be used to extract the DMI constant, *D*.[11]

Note that, without an in-plane field application, two DWs are symmetric and only a small reduction in coercive field can be observed due to the Joule-heating effect while there exists no hysteresis loop shifts.[11]

Supplementary Fig. 5 shows a summary of the DMI effective field measurement. The AH measurements were conducted at various in-plane dc currents (I_{dc}) and in-plane fields (H_x) to characterize magnetization switching as a function of I_{dc} and H_x. Exemplary AH loops at H_x = 25 mT (H_x = -60 mT) and I_{dc} = ±40 mA (I_{dc} = ±30 mA) are shown in Supplementary Fig. 5a (5b). At a fixed H_x, the AH loops are oppositely shifted for $I_{dc} > 0$ and $I_{dc} < 0$, implying the existence of current-induced effective field H_z^{eff} due to a spin Hall effect. It can also be found that, at a fixed I_{dc}, the AH loops are oppositely shifted for $H_x > 0$ and $H_x < 0$, indeed revealing that in-plane magnetic fields break the symmetry between *up-to-down* and *down-to-up* Néel DWs. Supplementary Fig. 5c,d plot the shifted center positions of AH loop, H_0, for in-plane fields of H_x = ±60 mT as a function of in-plane current, resulting in an estimate of H_0 $(=H^z_{eff})/I_{dc}$, which is the effective SHE-induced field; labeled as χ. By calculating the χ for a wide range of in-plane fields H_x, we can finally plot the χ as a function of in-plane field H_x as shown in Supplementary Fig. 5e. As was expected and also in good agreement with Ref. [11], χ increases up to H_x = ±190 mT, where it saturates, implying an estimate of effective DMI field being $|H_{DMI}|$ = 190 mT in our Pt/GdFeCo/MgO structure.

With the effective DMI field, the magnitude of DMI can be calculated by using $|D| = \mu_0 M_S \Delta |H_{DMI}|$ in Ref. [12], where Δ being the domain wall width, $\Delta = \sqrt{A / K_{u,eff}}$. By using material parameters obtained from VSM and assuming the exchange stiffness A = 10 pJ/m, the DMI constant can be calculated to be $|D|$ = 0.96 mJ/m^2. This value is in good agreement with other reported values using Pt-interfaced ferrimagnetic films in Refs. [13,14].

- **Magnetization compensation ratio, *n***

In order to evaluate the effective compensation ratio, $n = M_{S,FeCo} / M_{S,Gd}$, in our GdFeCo films at room temperature, we have performed the X-ray magnetic circular dichroism (XMCD) spectroscopy measurement at beamline 2A at Pohang Accelerator Laboratory (PAL), Pohang, Korea. The XMCD spectroscopy has been widely used to analyze element-specific magnetic properties in a ferromagnetic material.[15] Moreover, by integrating the areas of the absorption spectra of right- and left-circular polarized X-rays, one can calculate the spin and orbital magnetic moments quantitatively via *the sum rule.*[16–18] The sum rules are a powerful method to obtain the element-resolved spin and orbital magnetic moments especially for the magnetic

transition metals and their compounds.[18] We have measured the XMCD at the Fe and Co $L_{2,3}$ absorption edges in order to quantify the magnetic moment of each atom separately. However, it should be noted that the sum rules at the Gd $M_{4,5}$ absorption edges cannot be applied to the measurement of spin/orbital moments, due to the strong exchange between the *4f* and *5d* bands that alters the transition probabilities. Because the 4*f* electrons have strong Coulomb interaction with the 5*d* electrons, the XMCD intensities do not depend only on the outer shell (*5d* in this case) polarization, rather, the *4f-5d* exchange in rare earth material causes a spin split in *5d* band, thereby enhancing the transition probability for one spin channel.[18,19] Therefore, for measuring the magnetization compensation ratio, *n*, we first measured the spin moments of Fe and Co using the XMCD spectroscopy. Then, we numerically calculated the spin moment of Gd using the acquired $m_{\mathrm{s,Fe}}$, $m_{\mathrm{s,Co}}$ and the net magnetization per unit volume measured by VSM technique, enabling us to calculate the magnetization compensation ratio in the end.

Supplementary Fig. 6 shows the XMCD intensities, normalized by the averaged X-ray absorption spectroscopy (XAS) intensities, at the energies near Fe and Co $L_{2,3,}$ absorption edges. The difference between the XMCD spectra of right- and left-polarized light is also shown. By applying the sum rule to the acquired absorption spectra, we have calculated the effective spin and orbital magnetic moments per atom of Fe and Co as the following: $m_{\mathrm{s,Fe}} = 2.33\ \mu_B$, $m_{\mathrm{o,Fe}} = 0.26\ \mu_B$, $m_{\mathrm{s,Co}} = 1.54\ \mu_B$, $m_{\mathrm{o,Co}} = 0.19\ \mu_B$, where μ_B is the Bohr magneton. We confirm that the measured values are reasonable compared to their zero-temperature magnetic moments, $m_{\mathrm{s,Fe}}$ (0 K) = 2.2 μ_B, $m_{\mathrm{s,Co}}$ (0 K) = 1.7 μ_B.[20,21] We also note that the small amount of Co, ~9.4 at%, included in GdFeCo alloy may have caused the ill-defiled edges in XMCD spectra as shown in Supplementary Fig. 6, which may have resulted in relatively small value for Co effective spin moment. However, due to the small amount of Co, less than 10 at%, its effect on the overall result can be considered negligible.

Considering our 3-component (Gd, Fe, Co) system, where Gd and FeCo spins are aligned anti-parallel with Gd moment being larger than the FeCo moment ($M_{\mathrm{Gd}} > M_{\mathrm{FeCo}}$) below the magnetization compensation temperature, the saturation magnetization per unit volume can be expressed as follows[22]

$$M_S = N_a(X_{Gd}m_{s,Gd} - X_{Fe}m_{s,Fe} - X_{Co}m_{s,Co}) \quad (5)$$

where M_{S} is the effective net magnetization per unit volume, N_{a} is the number of atoms per unit volume, X_{i} is the atomic fraction of *i*-component within our GdFeCo alloy and $m_{s,i}$ is the spin moment per atom (units in μ_B per atom). The atomic fraction is given by the atomic

composition ratio of alloy target (Gd:Fe:Co = 25:64.6:9.4). The per atom spin moments for Fe and Co are calculated by the XMCD measurement ($m_{s,Fe}$ = 2.33 μ_B and $m_{s,Co}$ = 1.54 μ_B), and the effective net magnetization is measured from VSM, where M_S = 200 emu/cm^3. By adopting the analytical mean-field analysis of the magnetic properties proposed by A. Gangulee *et al.,*[22] the N_a value can also be estimated considering the facts that i) the volume per atom in metallic systems is almost independent of the structure[23] and ii) the density of crystalline materials decreases by ~5 % upon becoming amorphous[22] Thus, we estimate N_a as

$$N_a \approx \frac{0.95}{X_{Gd}v_{Gd} + X_{Fe}v_{Fe} + X_{Co}v_{Co}} \tag{6}$$

where v_i is the volume per atom of the *i*-th species, which can be calculated from their preferred crystalline structures (hexagonal-closed-packed structure for Gd and Co and body-centered-cubic structure for Fe) and empirical atomic radius. By putting the calculated N_a (= 4.6×10^{28} atom m^{-3}) and other known parameters into the Eq. (5), we calculated the effective spin moment of Gd in our GdFeCo being $m_{s,Gd}$ = 8.47 μ_B per atom, eventually leading to the compensation ratio of $n = M_{S,FeCo} / M_{S,Gd} = (X_{Fe}\times m_{s,Fe} + X_{Co}\times m_{s,Co}) / X_{Gd}\times m_{s,Gd}$ = 0.78.

- **Anisotropy and saturation magnetization**

VSM measurements shown in Supplementary Fig. 1 and Supplementary Note 1 yields a saturation magnetization being M_S = 2×10^5 A m^{-1} and an uniaxial anisotropy constant being K_u = 4.01 ×10^4 J m^{-3}.

- **Summary of experimentally measured material parameters**
  - Net saturation magnetization, M_S = 2×10^5 A m^{-1}
  - Uniaxial anisotropy, K_u = 4.01×10^4 J m^{-3}
  - Damping coefficient, α = 0.205 ± 0.035
  - Spin Hall angle, θ_{SH} = 0.055
  - DMI magnitude, D = -0.98 mJ m^{-2}
  - Compensation ratio, $n = (M_S^{Fe}+M_S^{Co})/M_S^{Gd} = 0.78$

Supplementary Note 4. Skyrmion diameter measurement.

Here, we explain the skyrmion diameter determination procedure used to extract the actual diameter of the individual skyrmion from scanning transmission X-ray microscopy (STXM) images. As shown in Supplementary Fig. 7, the diameter was determined by fitting four line-scans of the XMCD signal across the STXM image. By taking the average of full-width at half maximum (FWHM) of Gaussian fits, the skyrmion diameter was calculated by $D_{\mathrm{skyrmion}} = \sqrt{\left(D_{y=0}^2 + D_{x=0}^2 + D_{y=x}^2 + D_{y=-x}^2\right)/4}$, and the skyrmion diameter shown in Supplementary Fig. 7 is measured to be ~183 nm.

We have also performed micromagnetic simulations and measured skyrmion profiles with experimental parameters. Supplementary Fig. 8 shows the experimental and simulated profiles for the ferrimagnetic skyrmion. As shown in Supplementary Fig. 8, under a finite out-of-plane magnetic field (H_z = -140 mT), which is similar to the external field amount applied in experiments (H_z = -145 mT), the simulated skyrmion diameter and profile are in a good agreement with the observed experimental skyrmion profile. This agreement proves that the experimentally measured material constants are highly reasonable.

Supplementary Note 5. Electric connections and the actual pulse shape used for STXM measurement.

Supplementary Fig. 9a shows the electric connections used for the current-induced skyrmion behaviour measurement performed using STXM technique. Supplementary Fig. 9b shows an exemplary 5-ns-long pulse, used for skyrmion excitation, acquired using oscilloscope connected in the circuit. As described in the Methods in the main text, it should be noted that there exist only small amount of pulse loss due to the reduced impedance mismatch, R_{sample} (57 Ω) ~ R_{ideal} (50 Ω).

Supplementary Note 6. Static and dynamic properties of a ferrimagnetic skyrmion in the checkerboard-like two-sublattice spin system with classic J_1-J_2-J_2' Heisenberg exchange interactions.

Here, we study the static and dynamic properties of a ferrimagnetic skyrmion in the checkerboard-like two-sublattice spin system with both antiferromagnetic inter-sublattice (J_1) and ferromagnetic intra-sublattice (J_2, $J_2^{'}$) Heisenberg exchange interactions (see Supplementary Fig. 10). Supplementary Fig. 11 shows the size of the ferrimagnetic skyrmion as functions of the Gd-Gd and Fe-Fe intra-sublattice exchange interactions. It is found that skyrmion size decreases with increasing intra-sublattice exchange interactions. Supplementary Fig. 12 shows the velocity and skyrmion Hall angle of the ferrimagnetic skyrmion as functions of the Gd-Gd and Fe-Fe intra-sublattice exchange interactions. It can be seen that the skyrmion velocity decreases with increasing intra-sublattice exchange interactions, while the skyrmion Hall angle increases with increasing intra-sublattice exchange interactions.

However, it should be noted that, while we find that the intra-sublattice exchange interactions indeed affect on the ferrimagnetic skyrmion size and dynamics, the influence of these effects on the overall dynamics, especially on the skyrmion Hall angle, turns out not to be significant.

Supplementary Note 7. Supplementary simulation results on the skyrmion Hall effect in ferrimagnetic systems in various regime of damping coefficient and compensation ratio.

Supplementary Fig. 13a first shows the skyrmion velocity as a function of the driving current density in models with different values of the damping coefficient. The corresponding skyrmion Hall angle as a function of the driving current density for different damping coefficients is given in Supplementary Fig. 13b. It shows that the skyrmion velocity is proportional to the driving current density, while the skyrmion Hall angle is independent of the driving current density. Supplementary Fig. 13c and 13d show the skyrmion velocity and the skyrmion Hall angle as functions of the damping coefficient at a certain current density, respectively. It can be found that both the skyrmion velocity and the skyrmion Hall angle are inversely proportional to the damping coefficient.

Supplementary Fig. 14a,b,c shows the skyrmion velocity, the skyrmion Hall angle and the skyrmion size as a function of the compensation ratio n, respectively. As the value of compensation ratio approaches to 1, the net magnetic moment vanishes, implying that magnetic skyrmions change from ferromagnet to antiferromagnetic case. It can be seen that the skyrmion velocity, the skyrmion Hall angle, as well as the skyrmion radius decrease with increasing n in the given model. Moreover, it should be noted that skyrmion disappears when the compensation ratio is equal to and larger than 0.80, which is slightly larger than experimentally measured value, $n = 0.78$, implying that the internal dipolar field from further reduced effective saturation magnetization is not large enough to establish skyrmion phase with given material parameters.

Supplementary Note 8. Dynamics of ferrimagnetic and ferromagnetic skyrmions in the same clean and disorder models.

Supplementary Fig. 15 shows the skyrmion velocity and the skyrmion Hall angle of the ferrimagnetic and ferromagnetic skyrmions moving in the same clean and disorder models. In both the clean and disorder models, the velocity difference between ferrimagnetic and ferromagnetic skyrmions increases rapidly as the current density increases, implying that the mobility of ferrimagnetic skyrmion is much larger than that of ferromagnetic skyrmion (in agreement with Fig. 4 in main text). Moreover, in both the clean and disorder models, it is found that the skyrmion Hall angle of the ferrimagnetic skyrmion is significantly smaller than that of the ferromagnetic skyrmion regardless of the presence of pinning effect.

However, it is noteworthy that, when the driving current density increases from a small value (e.g. $j = 1\times10^{11}$ A m^{-2}) to a large value (e.g. $j = 3\times10^{11}$ A m^{-2}), the variation of the ferromagnetic skyrmion Hall angle is smaller than that of the ferrimagnetic skyrmion, which indicates a stronger Magnus force (i.e. the force leading to the skyrmion Hall effect) present in ferromagnetic material is helpful for the skyrmion depinning, which could force the skyrmion to make a detour when meeting a pinning site.

Supplementary References

1. Barker, J. & Tretiakov, O. A. Static and Dynamical Properties of Antiferromagnetic Skyrmions in the Presence of Applied Current and Temperature. *Phys. Rev. Lett.* **116,** 147203 (2016).
2. Zhang, X., Zhou, Y. & Ezawa, M. Magnetic bilayer-skyrmions without skyrmion Hall effect. *Nat. Commun.* **7,** 10293 (2016).
3. Kim, S. K., Lee, K.-J. & Tserkovnyak, Y. Self-Focusing Skyrmion Racetracks in Ferrimagnets. *ArXiv170202554 Cond-Mat* (2017).
4. Binder, M. *et al.* Magnetization dynamics of the ferrimagnet CoGd near the compensation of magnetization and angular momentum. *Phys. Rev. B* **74,** 134404 (2006).
5. Kittel, C. On the Theory of Ferromagnetic Resonance Absorption. *Phys. Rev.* **73,** 155–161 (1948).
6. Kim, S.-I., Seo, M.-S., Choi, Y. S. & Park, S.-Y. Irreversible magnetic-field dependence of ferromagnetic resonance and inverse spin Hall effect voltage in CoFeB/Pt bilayer. *J. Magn. Magn. Mater.* **421,** 189–193 (2017).
7. Flovik, V., Macià, F., Kent, A. D. & Wahlström, E. Eddy current interactions in a ferromagnet-normal metal bilayer structure, and its impact on ferromagnetic resonance lineshapes. *J. Appl. Phys.* **117,** 143902 (2015).
8. Beaujour, J.-M. L. *et al.* Ferromagnetic resonance study of sputtered Co|Ni multilayers. *Eur. Phys. J. B* **59,** 475–483 (2007).
9. Conca, A. *et al.* Low spin-wave damping in amorphous Co40Fe40B20 thin films: Journal of Applied Physics. *J. Appl. Phys.* **113,** 213909 (2013).
10. Liu, L., Moriyama, T., Ralph, D. C. & Buhrman, R. A. Spin-Torque Ferromagnetic Resonance Induced by the Spin Hall Effect. *Phys. Rev. Lett.* **106,** 36601 (2011).

11. Pai, C.-F., Mann, M., Tan, A. J. & Beach, G. S. D. Determination of spin torque efficiencies in heterostructures with perpendicular magnetic anisotropy. *Phys. Rev. B* **93,** 144409 (2016).

12. Thiaville, A., Rohart, S., Jué, É., Cros, V. & Fert, A. Dynamics of Dzyaloshinskii domain walls in ultrathin magnetic films. *EPL Europhys. Lett.* **100,** 57002 (2012).

13. Emori, S. *et al.* Spin Hall torque magnetometry of Dzyaloshinskii domain walls. *Phys. Rev. B* **90,** 184427 (2014).

14. Woo, S. *et al.* Observation of room-temperature magnetic skyrmions and their current-driven dynamics in ultrathin metallic ferromagnets. *Nat. Mater.* **15,** 501–506 (2016).

15. Schütz, G. *et al.* Absorption of circularly polarized x rays in iron. *Phys. Rev. Lett.* **58,** 737–740 (1987).

16. Thole, B. T., Carra, P., Sette, F. & van der Laan, G. X-ray circular dichroism as a probe of orbital magnetization. *Phys. Rev. Lett.* **68,** 1943–1946 (1992).

17. Carra, P., Thole, B. T., Altarelli, M. & Wang, X. X-ray circular dichroism and local magnetic fields. *Phys. Rev. Lett.* **70,** 694–697 (1993).

18. Stöhr, J. Exploring the microscopic origin of magnetic anisotropies with X-ray magnetic circular dichroism (XMCD) spectroscopy. *J. Magn. Magn. Mater.* **200,** 470–497 (1999).

19. Harmon, B. N. & Freeman, A. J. Spin-polarized energy-band structure, conduction-electron polarization, spin densities, and the neutron magnetic form factor of ferromagnetic gadolinium. *Phys. Rev. B* **10,** 1979–1993 (1974).

20. Schlosser, W. F. The temperature dependence of the magnetic moment of iron, nickel and invar for $T/T_c < 0.5$. *Phys. Lett. A* **40,** 195–196 (1972).

21. Srivastava, P. *et al.* Magnetic moments and Curie temperatures of Ni and Co thin films and coupled trilayers. *Phys. Rev. B* **58,** 5701–5706 (1998).

22. Gangulee, A. & Kobliska, R. J. Mean field analysis of the magnetic properties of amorphous transition-metal–rare-earth alloys. *J. Appl. Phys.* **49,** 4896–4901 (1978).

23. Barrett, C. S. & Massalski, T. B. *Structure of metals: crystallographic methods, principles, and data*. (McGraw-Hill, 1966).
